# Overweight among seafarers working on board merchant ships

**DOI:** 10.1186/s12889-018-6377-6

**Published:** 2019-01-09

**Authors:** Giulio Nittari, Daniele Tomassoni, Marzio Di Canio, Enea Traini, Isabel Pirillo, Andrea Minciacchi, Francesco Amenta

**Affiliations:** 1 Telemedicine and Telepharmacy Centre, University of Camerino, Via Madonna delle Caerceri, 9, 62032 Camerino, Italy; 2 School of Biosciences and Veterinary Medicine, University of Camerino, Via Gentile III Da Varano, 62032 Camerino, Italy; 3 Research Department, International Radio Medical Centre (CIRM), Via Dell’Architettura, 41, 00144 Rome, Italy

**Keywords:** Obesity, Overweight, Seafarers, Systolic blood pressure, Blood glucose

## Abstract

**Background:**

Obesity and overweight represent a relevant risk factor for seafarer’s health. The frequency and distribution of overweight and obesity among seafarers working on board of Italian flag ships were studied. Analysis was made on occupational medicine files collected, in the frame of health surveillance inspections, between 2013 and 2016 from Centro Internazionale Radio Medico (CIRM).

**Methods:**

The data of nationality, age, weight, height, blood glucose and blood pressure values obtained from 1155 seafarers were analyzed. Body mass index (BMI) values were calculated and compared with data reported for the general population of the same nationality of seafarers examined.

**Results:**

BMI values revealed a tendency to overweight, whereas blood glucose and systolic blood pressure values were in general in the normal range. Approximtely 40% of subjects investigated were overweight, and more than the 10% of them were obese. Underweight was noticeable only in 1.22% of crew members and 0.34% of officers. The 0.52% of subjects investigated was diabetic, and 2.68% were hypertensive. Seafarers, regardless their nationality and rank, showed a greater tendency to overweight and obesity compared with general population of the same ethnicity.

**Conclusions:**

Due to the occurrence of overweight and obesity among seafarers, campaigns for promoting awareness of the phenomenon and on the danger of these conditions for health should be promoted. Specific initiatives to avoid the assumption of junk food and the organization of adequate spaces, times and programs for physical exercise sessions on board should be offered for keeping seafarers healthier.

## Background

Overweight and obesity are very important issues in different countries, [1] mainly because these conditions are associated with other problems such as cerebrovascular and coronary diseases, and several other causes of death [2]. Obesity and metabolic syndrome are considered risk factors for dementia, and are associated with lower cognitive performance in population-based investigations [3–5]. Statistical studies conducted in 2014 indicated that 1.9 billion adults worldwide are overweight, and of the latter about 600 million are obese [6]. It is estimated that these statistics will increase in the coming years, especially in the United States [7] and in Europe [8].

The obesity condition develops when there is an excess of nutrients introduced, compared to those consumed, but the contribution of these factors is still quite misunderstood. Many studies have revealed that both the excess of energy intake and the reduction of energy expenditure can determine the onset of obesity [9, 10]. The body requires energy to support physiological functions, and when we introduce calories equal to the amount needed by the body, weight tends to remain the same. Over time people tend to eat and drink more calories than they burn, and excess calories lead to overweight, and progressively to obesity [11]. Obesity is a known risk factor for various diseases [12, 13] and can be considered a multifactorial pathology, due to genetic conditions [14], endocrine problems [15], impaired thyroid function, and environmental factors [16].

Obesity is recognized as a cause of physical inability among seafarers. In addition to the influence on health, being overweight may represent a safety issue on board ships. For example, performing emergency operations may be difficult for overweight people, such as taking emergency exits or climbing on a rescue boat. In this regard, it has been reported that fatal accidents are more common in shipping industry than in construction industry and manufacturing industry [17]. This suggests that, due to the difficult working conditions, seafarers should be fit for working on ships, to be able to face the most dangerous situations. Body mass index (BMI) and age are closely associated with work ability [18]. The report “Consultation on Obesity”, published in 1997, [19] by the World Health Organization (WHO) has reported an interesting system for classifying overweight and obesity. BMI system is used internationally [20] and is calculated by dividing the person’s body weight (in kilograms) with the squared height (in meters). Based on the WHO classification, normal conditions are BMI values between 18.5 and 24.9. Values between 25 and 29.9 indicate overweight, and values between 30 and 34.9 indicate an obesity condition. Moreover, values < 18.5 indicate an underweight condition [19].

Seafarers are a population at high risk of developing cardiovascular diseases and cancer [21]. For these workers, the ship is not only the working place, but a real living environment for quite long periods [22]. Many factors such as exposure to chemical substances, smoking, alcohol consumption and obesity increase the risk of developing tumors and cardiovascular diseases [23]. Several studies have shown that obesity and overweight are frequent conditions in seafarers [24, 25].

In this study we have evaluated the prevalence of overweight and obesity by calculating the BMI of seafarers working on Italian flag ships involved in long distance international routes, navigating the seas around the world. The purpose of this study was to investigate whether obesity can be considered as a risk factor for seafarers and to identify suitable corrective strategies. The data of BMI calculated in seafarers, were related to blood glucose and blood pressure levels, and compared to the values of general population of the same countries of the seafarers [26–29]. This information could give elements on us how life on board ships, characterized by easy access to food, determines a greater prevalence of overweight and obesity in seamen, especially in those coming from countries with more disadvantaged socio-economic conditions [30].

## Methods

This retrospective study is based on measurements made as a part of occupational medicine examinations that should be compulsory twice a year on Italian flag ships. Data examined for the present study were collected by the Centro Internazionale Radio Medico (CIRM), the Italian Telemedical Maritime Assistance Service (TMAS), in the frame of health surveillance activities performed on board ships. This study has analyzed 1155 medical records, carried out between 2013 and 2016 on seafarers on board of 20 Italian flag ships. All medical data (including identity of seafarers) are stored in the CIRM database and there are not accessible to externals. Data were extracted from the data base by the authors of this study. This study was made according in the frame of project called Health Protection and Safety on Board Ships (acronym: HEALTHY SHIP) [31].

From the medical examination reports, data on seafarers’ nationality, age, height, weight, blood glucose, blood pressure and the results of other basic medical tests were extrapolated. The seafarers receiving anti-hypertensive and hypoglycemic treatment were not considered in the statistical analysis (Table 1).

**Table 1:** Seafarers under pharmacological treatment

Pharmacological treatment	Nr	%
Arterial Hypertension Proparolol/Atenolol + Furosemide	35	3.03%
Portal Hypertension Nadolol + nitrate/ Nadolol	41	3.55%
Type II Diabetes Metphormine	97	8.39%
Diabetes+ Hypertension	6	0.52%
Total	167	14.45%

BMI values were calculated for each seafarer based on the anthropometric parameters reported in the medical records and classified according to the criteria proposed by WHO [19, 20]. In view of the heterogeneity of ethnicity of seafarers undergoing occupational medicine examinations, data obtained were compared with the national statistics of their respective countries, to assess whether the lifestyle of seafarers could affect their normal weight conditions. The BMI distribution was compared by the Ҳ^2^ test assuming data obtained from literature [26–29] as expected values.

The potential correlation between BMI with blood glucose and blood pressure levels was evaluated by comparing the data of these two parameters. The means of different parameters investigated were calculated from single subjects grouped per age or per rank and were expressed as means ± S.E.M. The significance of the differences between the mean values was analyzed by analysis of variance (ANOVA). The correlation between age and physiological parameters were calculated by Pearson’s test.

## Results

Analysis included 1155 medical records. All seafarers examined were male, aged between 21 and 66 years (mean 39.00 ± 11.38 years). As per nationality, 37% of them were Italians; 29% Indians; 22% from Philippines; 11% Romanians and 1% from other origins. The distribution of the BMI, blood glucose and systolic blood pressure values of seafarers divided for their rank on board Officers or non officers, e.g. crew members), are summarized in Table 2.

**Table 2:** Distribution of parameters investigated among seafarers grouped per rank

	No.	BMI Kg/m^2^	BLOOD GLUCOSE mg/dl	SYSTOLIC PRESSURE mmHg
Total	1155	25.7 ± 3.5	96.2 ± 11.7	118.1 ± 8.5
Officers	583	25.9 ± 3.5	96.1 ± 10.4	118.1 ± 8.5
Non officers	572	25.5 ± 3.5	96.3 ± 12.9	118.0 ± 8.4

The data are the mean ± S.D.

**Fig. 1:**
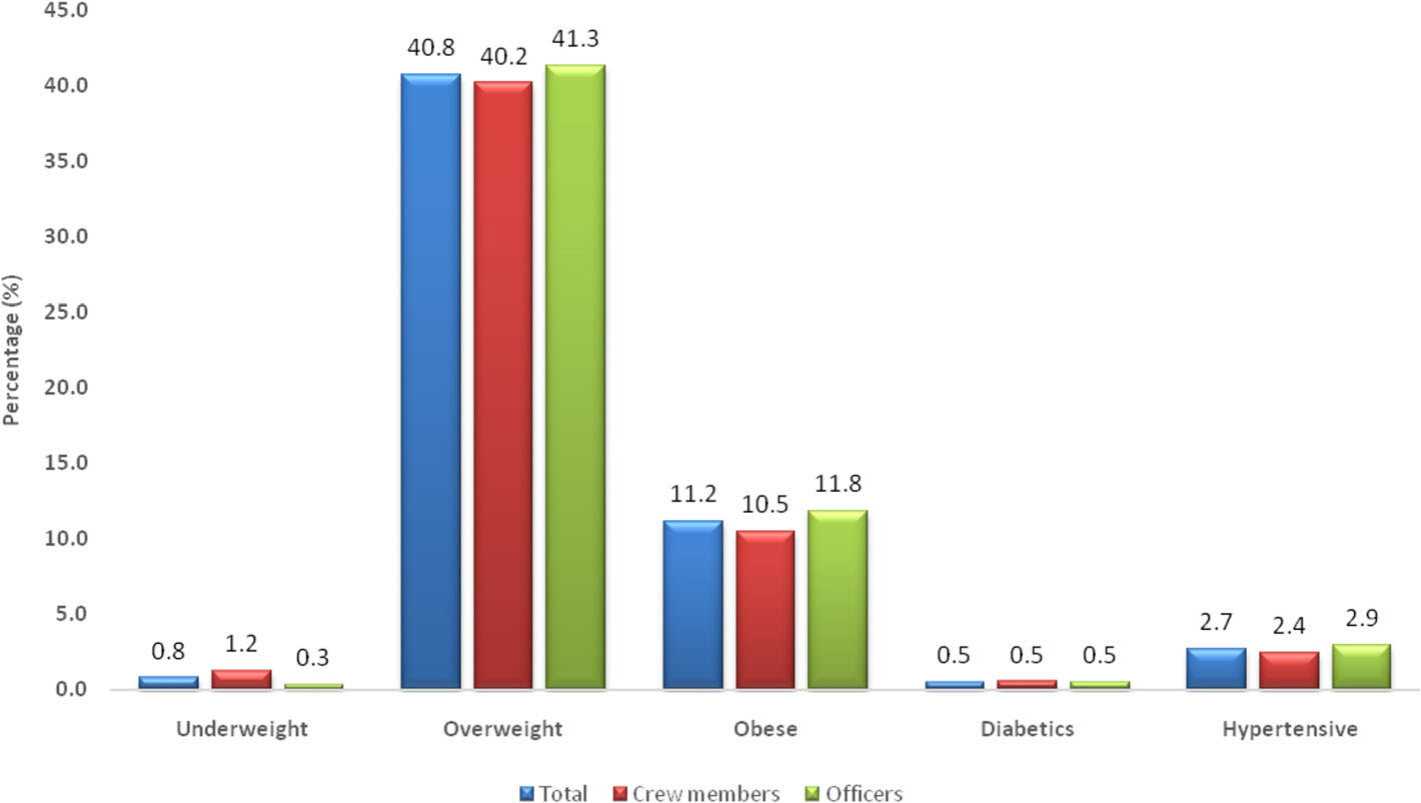
Percentage of seafears, divided in general by rank (officers and non-officers, e.g. crew members) whose parameters were not within normal limits

Data did not show differences between officers and crew members in all parameters considered. Mean BMI values showed a general tendency to the overweight condition, whereas blood glucose and mean systolic pressure values were in the normal range (Table 2). The percentage of subjects whose parameters were beyond normal limits is summarized in Fig. 1.

Over 40% of all subjects examined (officers or non-officers) resulted overweight, and over 10% (10.49% crewmembers and 11.84% officers) were obese. Only 1.22% of crew members and 0.34% of officers resulted underweight (Fig. 1). Only 0.52% of subjects examined were diabetic (0.52 crew members, 0.51% officers), and 2.68% (2.45% crew, 2.92% officers) resulted hypertensive. No significant differences were found between the two rank groups considered.

**Fig. 2:**
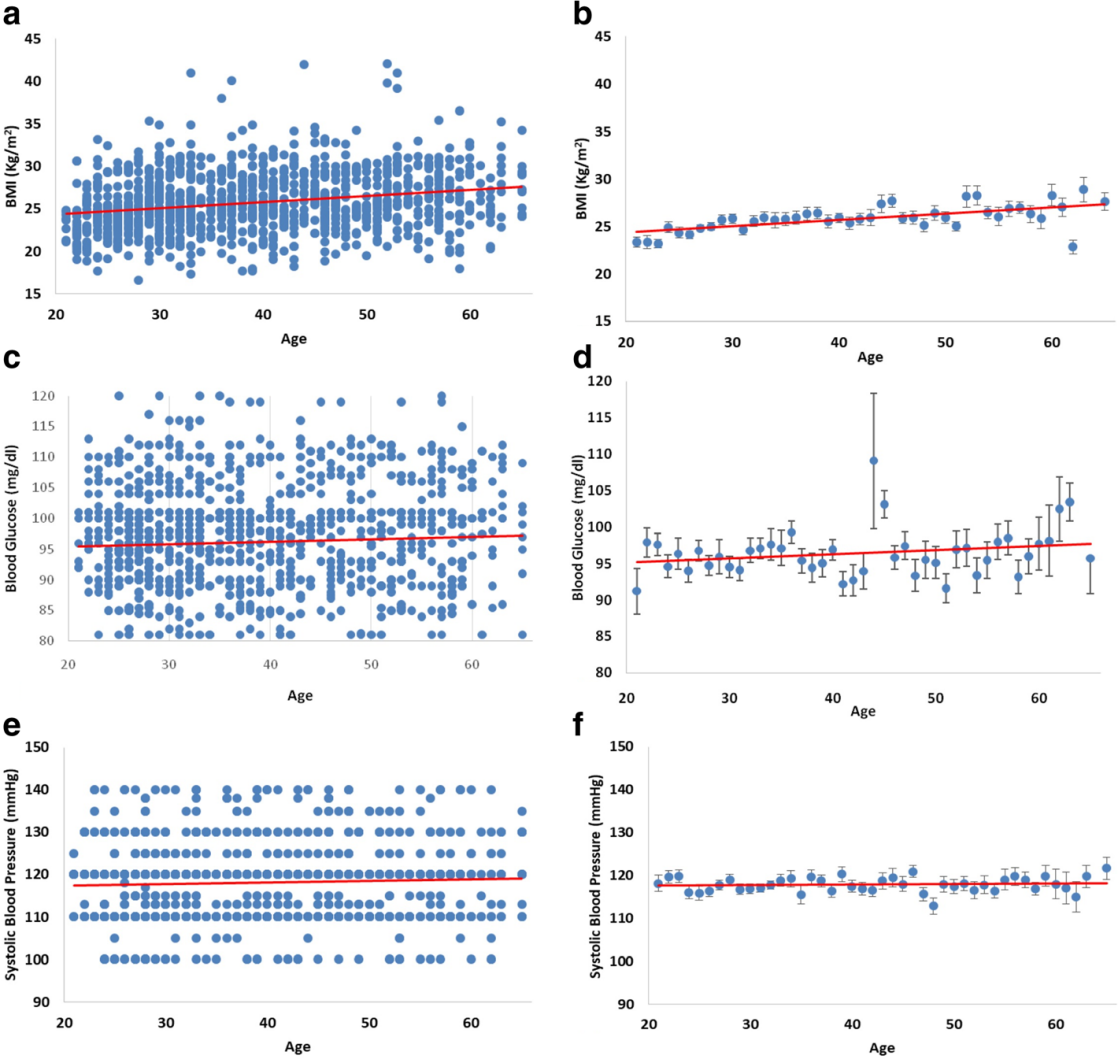
**a** Scatterplot of body mass index (BMI Kg/m^2^) values by age of subjects examined. **b** Mean ± S.E.M of BMI values for age. **c** Scatterplot of blood glucose (mg/dl) values by age of subjects examined. **d** Mean ± S.E.M of blood glucose values for age. **e** Scatterplot of systolic blood pressure (mmHg) values by age of subjects examined. **f** Mean ± S.E.M of systolic blood pressure values for age

The distribution of BMI, and the mean values for age are summarized in Fig. 2 a and b. A direct correlation was found between these two parameters (Table 3), with an increase of body weight with age mainly in subjects over 45 years. The same distribution was found both in crew members and officers (data not shown). Blood glucose (Fig. 2 c and d) and systolic blood pressure (Fig. 2 E and F) values were independent from aging as confirmed by Pearson’s correlation (Table 3).

**Table 3:** Pearson correlation coefficients and significance between age and physiological parameters examined

	Pearson Correlation	Significance
BMI		
Total	0.23#	8.88*10^−16^
Crew members	0.24#	4.33*10^−9^
Officers	0.25#	1.94*10^−9^
Blood glucose		
Total	0.04	0.21
Crew members	0.02	0.70
Officers	0.06	0.12
Systolic pressure		
Total	0.05	0.11
Crew members	0.03	0.43
Officers	0.06	0.13

# = *p* < 0.05 in Pearson’s test

Analysis of the correlations between age and the physiological parameters investigated by the Pearson’s test, is shown in Table 3, whereas the correlation between BMI and blood glucose levels and systolic pressure levels is summarized in Table 4. Blood glucose levels slightly correlated with the BMI values, whereas systolic blood pressure values were independent from BMI (Table 4).

**Table 4:** Pearson correlation coefficients and significance between BMI and physiological parameters examined

	Pearson Correlation	Significance
Blood glucose		
Total	0.14#	1.24*10^−6^
Crew members	0.12#	0.003
Officers	0.17#	4.65*10^−5^
Systolic pressure		
Total	0.01	0.85
Crew members	0.01	0.81
Officers	8.20*10^−4^	0.98

# = *p* < 0.05 in Pearson’s test

Data obtained were further analyzed and referred to the nationality of the seafarers. BMI values calculated for seafarers were also compared with those reported in the general population of the same ethnicity (country) groups.

The comparison of mean BMI value between seafarers and onshore population of the same nationality (from obtained WHO database) revealed differences for Filipino and Indian populations. Filipino seafarers averaged a BMI of 24.7 whereas the same value in Filipino general population was 22.6. The same is true for our Indian seafarers with a BMI of 25.7 seafarers vs 21.5 onshore. Romanian seafarers had a BMI of 27.2, whereas this value for general population averaged 26.9. For Italians, BMI values were similar to the population of the respective countries (25.8 seafarers vs 26.9 onshore). The prevalence of seafarers in the different weight classes was also compared to the values of the population of the respective countries. As shown in Table 5, the percentage of overweight and obese subjects was higher in seafarers compared to the general population of the same country (Table 5). The Filipino seafarers examined in this study were more overweight (30.4% versus 17.9% of the general population) or obese (7.4% versus 3.0% of the general population) compared to the population of compatriots. [26] 41.2% of Indian seafarers were overweight and 11.8% of them were obese. These values are higher than those of the respective male population, accounting for 8.4% of overweight and only 1.3% of obesity. No significant differences in the values of overweight were noticeable in Italian seafarers compared to the Italian general population (Table 5), although among seafarers a slightly higher percentage of obesity compared to general population was noticeable (Table 4). In Romanian seafarers, overweight and obesity were similar than in the general population (Table 5). The differences in BMI distribution of seafarers compared to those of general population were statistically significant at the Χ^2^ test (*p* < 0.05) for Filipino, Indian and Romanian seafarers.

**Table 5:** Body mass index percent distribution among seafarers of different nationalities compared with general population data of the same country

	Underweight (< 18.5)	Healthy Weight (18.5–24.9)	Overweight (25–29.9)	Obese ≥ 30	Overweight & Obese
Filipinos general population mean [25]	10.6%	68.5%	17.9%	3.0%	20.9%
Filipinos seafarers	1.2%	61.1%	30.4%	7.4%	37.7%
Indian general population mean [26]	33.7%	56.6%	8.4%	1.3%	9.7%
Indian seafarers	0.9%	46.2%	41.2%	11.8%	52.9%
Italian general population mean [27]	0.8%	50,9%	39.8%	8.5%	48,3%
Italian seafarers	1.6%	46.4%	40.6%	11.4%	52.0%
Romanian general population mean [28]	0.9%	29.1%	53.1%	16.9%	70.0%
Romanian seafarers	0	23.1%	58.7%	18.2%	76.9%

## Discussion

This study has shown the occurrence of overweight and obesity among seafarers examined on board of Italian flag ships. Moreover, we have observed that male seafarers working on board Italian merchant ships gain excessive weight around the age of 39–45 years, and reach the highest BMI in the group of 55–66 years of age. The present epidemiological analysis was performed on a particular category of workers, the seafarers, whose life styles undergo significant conditioning, due to the fact that they work on board of ship for months.

Analysis of the physiological parameters of blood glucose and systolic blood pressure levels did not show a direct correlation with age. Although 2.68% of the subjects was diabetic and 0.52% hypertensive, the non-correlation between body weight increase, blood glucose levels and hypertension, would exclude a condition of metabolic syndrome in the seafarers examined. On the other hand, no significant differences in terms of overweight and obesity were found between officers and crew. In fact, 41.44% of officers and 40.21% of crew members were overweight and 11.84% of officers and 10.49% of crew were obese. Despite the significant number of subjects studied in this work, it was not possible to correlate the results to the educational levels, the socio-economical conditions or to the physical activity, as these data are not considered in occupational medicine screenings of seafarers. Hence, these data were not available in the patients medical records.

Comparison of our data on excessive weight among seafarers embarked on Italian ships with the results of other studies (26–29) showed an undesirable weight pattern among seafarers, with a higher tendency to overweight and obesity in this category of workers.

The 254 Filipinos seafarers analyzed in this study showed a percentage of overweight and obesity respectively of 30.4% and 7.4%. In the Filipino adult general population, over the age of 20 years, percentages of overweight and obesity were respectively 17.9 and 3.0%. Filipino seafarers with a BMI > 25 kg / m^2^ account for the 37.7% of the sample compared to 20.9% of the Philippines general population [26]. Out of the 335 Indian seafarers esamined in the study, 41.2% was overweight and 11.7% obese (Table 5). In the Indian adult general population (15–54 years), an 8.4% of overweight and 1.3% of obese individuals were reported. Indian seafarers who have a BMI > 25 kg/m^2^ are 52.7%, and therefeore they account for a significantly higher proportion compared to 9.7% of males of general population estimated by a study conducted by the International Institute for Population Science [27].

Italian seafarers with a BMI > 25 kg/m^2^ were 40.6% of the total, compared to 39.8% of the Italian population. In contrast, obese Italian seafarers were more compared to the Italian male population [28]. Romanian seafarers with a BMI > 25 kg / m^2^ were 58.7% of the total, compared to 53.1% of the Romanian general population. Seafarers of the same nationality with a BMI > 30 were 18.2%, whereas obese individuals in the Romanian male general population averaged the 16.9% [29].

Overweight and underweight values in seafarers compared with those of the general population were higher in people living in lower income countries. A possible explanation of this observation is that the abundance of food on board stimulated exaggerated eating as a sort of rewarding versus a more limited availability in home countries. This excessive eating promotes overweight. This relationship is less obvious for obesity indicating that obesity has more complex causes than the simple overweight that could be promoted just by excessive eating. This hypothesis is supported indirectly by the observations that in Italian seafarers, coming from the country with the highest per capita income among the four analyzed in this work [30], no relevant differences in overweight and obesity percentages compared with general population were noticeable.

## Conclusion

Our data show an increased tendency of being overweight and obese among seafarers, compared to the general population of the same ethnicity. This condition may be due to unhealthy lifestyles such as inappropriate diet, lack of fresh food in the diet, consumption of large quantity of sugared tea, coffee and beverages because of their odd working hours and unique lifestyle, lack of physical activity. Unfortunately, no data on diet and physical activities can be obtained from the electronic health records analyzed in this study. This prompted us to develop a specific lifestyle questionnaire that will be administered to seafarers, to obtain useful information about their lifestyle.

Other studies confirm the high incidence of overweight and obesity in American, Croatian and Danish seafarers. [32–34] In this context, the analysis carried out on a group of mariners revealed that about 80% of them is not satisfied with the quality of food available on read board and would like to eat healthier; 20% of them retain food in their cabin and approximately 20% of these seafarers use dietary supplements to overcome dietary gaps. Fresh products are also not often available on board [35].

During sea voyages, seafarers have no choice in terms of quality of food, and meals are influenced by the presence of different ethnicities [36]. Other studies in literature suggest that inappropriate nutrition on board is a widespread problem [37, 38]. A survey based on the eating habits of Chinese seafarers showed a shortage of vitamin C, vitamin B2, vitamin A and calcium based on a daily diet [39].

In general, seafarers have also insufficient levels of physical activity. A study carried out on a Norwegian group of seaman showed that 70% of them practiced physical exercise at home twice a week, whereas only 39% were used to training on board. Moreover, 20% never performed physical exercise on board while only 5% of the sample did not practice sports even at home [40]. On the other hand, with the evolution of marine technology and equipment, onboard work is largely sedentary and requires minimal physical effort [41].

In view of the occurrence of overweight and obesity among seafarers, campaigns for promoting awareness of the phenomenon and on the danger for health of these conditions should be promoted. Specific initiatives to avoid the assumption of junk food, preferring healthy foods, ensuring fresh food supplies, adequate intake of vitamins and mineral salts, and regularizing meals on board should be undertaken. Organization of adequate spaces, times and programs for physical activity on board ships should also be offered for keeping seafarers healthier. Promotion of a correct and healthy lifestyle can reduce the incidence of overweight and related pathologies that likely will appear after the service on board ships (e.g. cardiac and cerebrovascular diseases). Management of overweight-related pathologies in an environment such as a ship at high sea may be difficult, taking into account that merchant ships have no physicians or expert health professionals on board as well as they are provided with limited medical facilities [42]. In view of this, prevention more than treatment of pathologies is the most reasonable strategy to pursue for protecting the health of a category of workers which, in general, receives less health protection than workers ashore.

## Abbreviation

BMI: Body Mass Index
CIRM: Centro Internazionale Radio Medico
TMAS: Telemedical Maritime Assistance Service
WHO: World Health Organization
